# Evolution of the major alcohol companies key global policy vehicle through the prism of tax records 2011–19

**DOI:** 10.1186/s12992-023-00933-w

**Published:** 2023-05-25

**Authors:** Matthew Lesch, Jim McCambridge

**Affiliations:** Department of Health Sciences, Faculty of Sciences Area 4, A/TB/212, University of York, Seebohm Rowntree Building, Heslington, YO10 5DD York UK

**Keywords:** Alcohol policy, Alcohol industry, Global public health, Commercial determinants of health

## Abstract

**Background:**

Important insights have been generated into the nature of the activities of the International Center for Alcohol Policies (ICAP). Its successor, the International Alliance for Responsible Drinking (IARD) is less well understood. This study aims to rectify evidence limitations on the political activities of the alcohol industry at the global level.

**Methods:**

Internal Revenue Service filings were examined for ICAP and IARD each year between 2011 and 2019. Data were triangulated with other sources to establish what could be gleaned on the internal workings of these organisations.

**Results:**

The stated purposes of ICAP and IARD are near identical. The main declared activities were similar for both organisations and comprised public affairs/policy, corporate social responsibility, science/research and communications. Both organisations work extensively with external actors and it has become possible more recently to identify the main contractors supplying services to IARD.

**Discussion:**

This study sheds light on the political activities of the alcohol industry at the global level. It suggests that the evolution of ICAP into IARD has not been accompanied by shifts in the organisation and activities of the collaborative efforts of the major alcohol companies.

**Conclusion:**

Alcohol and global health research and policy agendas should give careful attention to the sophisticated nature of industry political activities.

## Introduction

The alcohol industry is a significant player in the global economy. In 2020, the industry was valued at $1.49 trillion (USD) [1]. The size of the alcohol market has significant implications for global health. Alcohol consumption is estimated to contribute causally to about 3.3 million deaths each year [2]. In the past 25 years, producers have expanded so that they produce and promote brands across continents [3, 4]. The top ten producers now account for the majority of worldwide consumption of beer and spirits respectively and have strategically targeted low- and middle-income countries and regions for expansion [4]. The concentrated nature of the market has led to the creation of organisations explicitly tasked with coordinating the political activities of the major alcohol producers [5].

Several theoretical frameworks have been developed to understand the nature of corporate political activities [6], particularly in the context of unhealthy commodity industries (UCIs), including alcohol and tobacco [7–9]. According to these frameworks, several structural, institutional and ideological conditions provide key opportunities for industry to influence those with decision-making power. Alcohol researchers have identified key strategies that industry uses in pursuit of its political goals. These include lobbying [10–17], framing [18–27], litigation [28–32], and corporate social responsibility (CSR) initiatives [33–43]. Industry has also funded research studies in an effort to influence the content of scientific research and such evidence is used in policy making [35, 44–54]. The alcohol industry has long exerted influence over the shaping of ideas related to alcohol, alcohol problems, alcohol policy, and the alcohol industry itself. This influence is aimed at ensuring that these strategies operate in synergy with one another [55].

Little is known, however, about the organisations that lead the alcohol industry’s political activities, particularly at the global level (though, there are recent exceptions, for example [56]). Similar to other industries [57, 58], trade associations are often the key vehicles for coordinating industry actors’ political activities. Trade associations can mobilise different actors from across a single sector (for example, alcohol) or within a specific segment of the sector (for example, production or retail) [59]. Social aspects public relations organisations (SAPROs) are distinct from trade associations in that they are primarily designed to advance the alcohol industry’s CSR goals [34, 38, 42]. These organisations are “outwardly established to reduce alcohol-related harms” [60] but their core function is “to manage issues that may be detrimental to [the alcohol industry’s] interests, particularly in areas that overlap with public health” [61]. While SAPROs share some key similarities with tobacco industry front organisations [37, 62], these organisations are specific to the alcohol industry. The International Alliance for Responsible Drinking (IARD) is the only SAPRO operating globally. Most SAPROs, such as Drinkaware in the UK, are organised at the national level.

There are studies of IARD’s predecessor, the International Center for Alcohol Policies (ICAP) [45, 63–66]. One key study analysed publically available material produced by ICAP, including policy papers, conference proceedings, and tax filings. According to this investigation, ICAP engaged in several key activities, including lobbying, promoting collaborations between public health researchers and industry, and creating a parallel scientific literature on alcohol. ICAP’s main aim was to challenge the World Health Organization’s (WHO) position as the pre-eminent voice on alcohol policy issues [45]. A range of alcohol industry actors, including ICAP, have also been highly active in alcohol policymaking processes at the domestic and global level [21, 56, 59]. Moreover, only a few jurisdictions have embraced the WHO’s “best buys” policies [67] – that is, tougher restrictions on alcohol pricing, promotion and availability [68] (though, there are key recent developments in several countries, including Scotland, Ireland, and Lithuania) [69–71]. Yet the precise programs developed by ICAP to influence policy have not been adequately described or analysed.

IARD was formed in 2014 out of a merger of ICAP (which had been created by several alcohol producers in 1995), and the Global Alcohol Producers Group, a trade association [72]. Compared to ICAP, IARD’s actions have hardly been studied at all [21], indicating that little is known about a potential threat to global health.

One persistent challenge in analysing alcohol industry activity, including organisations such as ICAP and IARD, is the paucity of data relating to internal operations [59]. This study aims to deepen understanding of the alcohol industry’s political activities at the global level. Specifically, it seeks to enrich understanding of alcohol industry activity at the global level through careful description and analysis of ICAP and IARD’s tax filings. Analysis of tax records, in the context of other ICAP and IARD data, can help address the deficit in understanding how these organisations have operated, the nature of their relationships with each other, and potentially yield insights into the political organisation of the alcohol industry.

## Methods

We began with a scoping exercise to identify potential data sources. This included examining IARD’s website and references to IARD on the individual member companies’ (see below) websites for triangulation and analytic purposes. Informed by the earlier work [45], we collected a later set of ICAP and IARD’s Internal Revenue Service (IRS) tax documents between 2011 and 2019. Non-profit organisations in the US are required to submit an IRS Form 990 every year. The disclosure of detailed information, including on key programs and expenditures, provides a key potential data source for understanding the internal operation of US-based SAPROs.

We searched for and collected ICAP and IARD’s tax documents using ProPublica’s Nonprofit Explorer database (https://projects.propublica.org/nonprofits). ProPublica houses public documents, including federal tax filings, which can shed light on issues pertinent to the public interest. Using the search term “International Alliance for Responsible Drinking”, we retrieved tax filings for both ICAP and IARD from 2011 to 2019. ML reviewed these filings (ICAP, 2011–14 and IARD, 2015–19) and produced annual data summaries.

We used thematic analysis to guide data collection and analysis [73]. ML placed the tax documents into NVivo. To identify main expenditures (i.e., programs) over time, ML inductively generated several thematic codes. The coding approach was informed by previous research on ICAP [45], IARD [43], and alcohol policymaking [59]. Specifically, particular attention was paid to external-facing activities, including lobbying, stakeholder engagement, and research. ML compiled and analysed relevant financial information, including expenditure data. Finally, ML and JM placed these data in the context of what was known about these organisations in other cited data sources.

## Results

### Organisational characteristics and declared purposes

ICAP was created in 1995 by ten of the largest beer and spirits companies (see Appendix). ICAP’s professed organisational aim, as stated on its website in 1998 and its 2011 tax filing, was: “to help reduce the abuse of alcohol worldwide and promote understanding of the role of alcohol in society. To encourage dialogue and pursue partnerships involving the beverage alcohol industry, the public health community and others interested in alcohol policy” [74, 75].

In October 2014, ICAP announced that it would transition to a different organisation, IARD, with the new CEO starting in January 2015 [72]. IARD has described its core mission as “[t]o reduce harmful drinking and promote responsible drinking” [76–79]. In 2019, the organisation included the additional text: “Also to encourage dialogue and partnership involving beverage alcohol industry, the public health community and others interested in alcohol policy” [80]. According to its first press release, IARD would “advocate for the most effective policies and programs, communicate the views and perspectives of member companies, and serve as a single global point of contact for international and national agencies, member states, NGOs, and other stakeholders” [72]. One aspect of the metamorphosis is that ICAP’s claims of independence from member companies [45] have been jettisoned, as IARD explicitly presents itself and its activities as CSR for the companies [81, 82].

According to member companies’ materials, a key function of IARD is to collaborate and thereby serve to coordinate the producers’ political activities. According to Beam Suntory, through IARD, “leading beverage alcohol producers put [their] commerical competitiveness aside to create a shared force in [their] Global Commitments to reduce alcohol miuse” [83]. Other producers provide more nuance, outlining more explicitly policy-orientated goals. Carlsberg portrays IARD as a mechanism for promoting “industry-wide discussions and actions… [and] to secure joint lobbying efforts and voluntarily develop industry codes of conduct” [84]. Although IARD’s described role varies across member companies, CSR and related policy functions are apparent.

IARD’s website offers material on its internal structure. The Board of Directors comprises one representative from each member company and is led by a chair that serves a two-year term. The board members come from several departments within companies, including legal and corporate affairs and government relations. The IARD Board meets three times per year [85]. Senior staff execute the key functions, including approval of its tax filings. IARD was led from 2017 by Henry Ashworth (CEO), the former head of the Portman Group, a key alcohol producer association in the UK [80]. Earlier Marcus Grant served as CEO throughout the ICAP years, then Ann Keeling led IARD (2015–16).

### ICAP’s key activities as identified in the tax filings

IRS 990 forms stipulate that organisations must disclose and describe their most significant programs (as measured by expenditure). Table 1 summarises ICAP’s programs.

**Table 1:** Major ICAP activities declared

Program	Description
Global actions on harmful drinking	A set of CSR-related initiatives “dedicated to helping reduce the harmful use of alcohol” in 18 countries. The program focused on three areas: drink driving, self-regulation and noncommerical alcohol” [75]
Public Affairs/Policy^a^	Engagement with identified institutions and opinion leaders, monitoring policy and offering national policy guidance to ICAP members. In targeting the WHO specifically, ICAP sought to “to encourage a more balanced approach to alcohol policy” [75]
Research^b^	Supported research projects were conducted “by outside scientists working independently or in cooperation with ICAP staff.” The main substantive areas of research focus were “unrecorded/non-commercial alcohol, education and quality of life” [86]
Communications^c^	ICAP’s main communication activities focused on website development, media relations, and “promoting the uptake and utilization of the organization’s technical materials” [75]

^a^The Public Affairs/Policy umbrella category was created by the authors to capture several similar smaller programs

^b^The Research category was generated by the authors after combining the major research activities

^c^The Communications umbrella category was created by the authors after identifying several similar smaller programs

The most significant program for ICAP between 2011 and 2014 was Global Actions on Harmful Drinking (GAHD) [75]. The GAHD was a set of CSR initiatives agreed to by the CEOs of major alcohol companies, with a focus on low- and middle-income countries, such as China, Russia, and Nigeria. GAHD was framed as an effort by industry “to encourage responsible drinking and discourage excessive or irresponsible drinking” [87]. The initiatives included 1) self-regulation: developing alcohol marketing codes of conduct) 2) drink driving: providing capacity-building and training tools, and 3) non-commercial alcohol: “measuring the informal alcohol market in nine different countries” [88]. ICAP was tasked with overseeing implementation.

Public affairs constituted another major ICAP activity. ICAP’s 2011 and 2012 tax records refer to the WHO Strategy. The purpose of this program was “to facilitate private sector activities in support of the objectives of the WHO Global Strategy to Reduce the Harmful Use of Alcohol” and to “report these activities to the international community” [75]. ICAP identifies regular engagement with “key opinion leaders” from the WHO, EU, UN and unspecified NGOs as central to its public affairs strategy. In 2011, for example, ICAP worked “to intensify dialogue with the [WHO]” [75]. ICAP was not only focused on WHO officials in Geneva but also sought to “develop links with [WHO’s] regional offices” [89]. The 2014 tax records offered insight into these efforts, explaining how IARD “monitored policy” for its members and supplied information about regulatory issues across different jurisdictions. IARD also offered “national policy guidance”, providing “reports for industry on WHO and EU developments… as well as… developments in Africa and Latin America regions” [79].

ICAP also engaged in several research-related activities. The tax filings provide some insight into the substantive focus of that work. This “research” included developing new quantitative measures to capture “the contribution of drinking to wellbeing and quality of life” and reviewing the evidence “on the relationship between drinking and psychosocial benefits” [75]. ICAP also promoted the “uptake and utilization” of this work in the broader research community and beyond, particularly its book, Working Together to Reduce Harmful Drinking [90]. It had also earlier published an Alcohol and Society Book Series, whose contents were produced by researchers and industry actors [45] along with ICAP staff. Finally, ICAP identified “conflict of interest” as a priority area. ICAP was interested in identifying “sources of potential conflict of interest around public health and policy issues” and developing a “framework” for addressing such conflicts [75].

ICAP also focused on communications to its members and stakeholders. This included reports summarising the “scientific evidence on health and policy issues relating to alcohol” which were supplied to governments, public health actors, and other stakeholders [86]. ICAP also developed training programs for industry stakeholders. These included online resources to explain “key policy and social aspects issues” for industry actors [75]. For 2014, ICAP or IARD also described efforts to “enhance [its] digital footprint through social media strategy” [79].

### IARD’s key activities

**Fig. 1:**
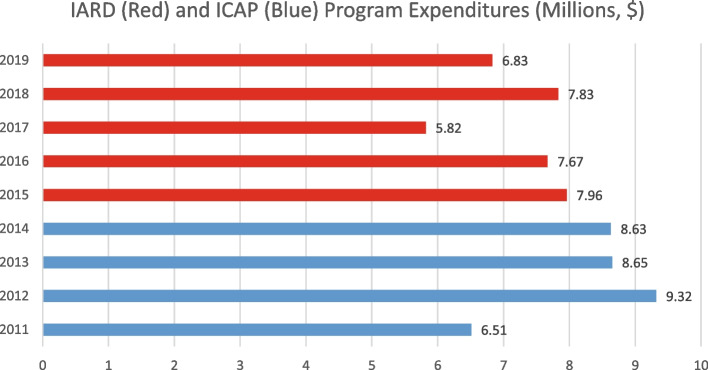
ICAP (2011–14) and IARD’s (2015–19) major program expenditures

IARD’s average annual spending on programs has been lower than its predecessor (see Fig. 1). Although ICAP existed for most or all of 2014 and IARD did not, the submission made for this year referred to IARD. We’ve included these data as ICAP.

IARD’s main programs are characterised by similar activities as ICAP (see Table 2). One of IARD’s key programs centred on implementing the Producers’ Commitments. IARD served as the secretariat for these commitments, which involved coordinating “reporting on the commitments” and publishing annual reports [77, 91, 92].

**Table 2:** Major IARD activities declared

Activity	Description
Producers’ Commitments	At an international conference in October 2012, the largest global producers of beer, wine and spirits agreed to implement the five following CSR-related objectives between 2013 and 2017: 1. Reducing underage drinking 2. Strengthening and expanding marketing codes of practice 3. Providing consumer information and responsible product information 4. Reducing drinking and driving 5. Working with retailers to reduce harmful drinking IARD was later charged by the signatories with implementation and monitoring these CSR initiatives [93, 94]
Science and policy	An “international scientific” program focused on better understanding “the relationship between drinking and health and social outcomes.” The research program included the development of “literature and materials” which were used to inform members and stakeholders [77] Like ICAP, the program “supported research by outside scientists working independently or in cooperation with the center staff.” Its main research topics included “unrecorded alcohol, education and quality of life” [78]
Communications	IARD communicated with stakeholders “through multiple channels, including meetings, website resources, electronic publications, and social media” [78] One of the main communication activities was to promote its “key reports and events to the global and regional media” [76]
Public Affairs	The aim of this IARD program was “to increase understanding of the global alcohol policy arena and various related topics in relevant multilateral fora” [76, 77]. This involved tracking alcohol policy developments at the global, regional and national levels, which allowed it to provide producers with “regular updates on these issues” [77]

The labels in the “Activity” column were developed by IARD. In the case of Producers’ Commitments, however, IARD referred to this set of initiatives as “Program Development” in its 2015 tax filing. Between 2016 and 2018, this activity is described as “Producers’ Commitments”

Between 2015 and 2019, “science and policy” was another major IARD program. IARD’s description of this program changed somewhat between tax years. In 2016, for example, IARD refers to “original research” and plans for this material “to be published.” Yet between 2017 and 2019, there is no mention of IARD’s original research or dissemination plans [76, 77, 80]. It appears IARD has also turned to external scientists, as was the case with ICAP. IARD’s use of other “consultants” may also be relevant (see below).

IARD uses a range of communication tools to promote itself and its activities, including a website, in-house reports, and media relations. As noted above, tax records from the latter years provide less detail about the nature of some activities, including communication activities [76–78, 80].

IARD’s public affairs program is also portrayed in vague terms, particularly so in more recent years. In comparing filings, ICAP provides much greater insight into the nature of the program. This is in keeping with the style of IARD’s website compared to that of ICAP. For example, authors of IARD documents are not identifiable, except where ICAP materials are presented. ICAP’s last tax filing in 2014 provides some insight into how relationship-building with WHO officials operated. IARD explains how it provided WHO headquarters with valuable data on alcohol “production and consumption” [79].

### IARD’s Involvement with third parties in 2018–19

IARD’s tax records from 2018 and 2019 offer contractual data on several agencies, including consultancies. Table 3 summarises this data, with contractors listed in order of the total size of their IARD contracts.

**Table 3:** List of IARD contractors, 2018 and 2019

Contractor	Purpose	Location of consultancy	Amount
Wilton Park Executive Agency UK’s Foreign Office)	Multi-stakeholder consulting	London	$398 k (2018) $400 k (2019)
Stiehle Consulting	Consulting services	Geneva	$398 k (2018) **$**389 k (2019)
World Federation of Advertisers	Marketing consulting	Brussels	$281 k (2018) $198 k (2019)
MSL Group London Ltd	Public Relations and Communications	London	$244 k (2018)
MM Science and Policy Advisors	Consulting services	Washington DC	$145 k (2019)

Wilton Park is an executive agency of the UK Foreign Commonwealth and Development Office which specialises in facilitating international policy discussions. According to its website, Wilton Park organised several alcohol policy discussions across the Caribbean, Central and South America, and Sub-Saharan Africa in 2019. The aim was to “build consensus and common purpose on how alcohol producers can best support global efforts to improve health and reduce the harmful use of alcohol” [95].

Both the 2018/19 records refer to a public affairs firm called Stiehle Consulting. Stiehle Consulting’s remit is to advise “clients on the role of Geneva-based international organizations, UN specialized agencies, commissions and programmes, and non-governmental organizations in international policy making”, including the World Health Organization. According to its website, Stiehle Consulting identifies “opportunities for interaction”, provides “information, insights and intelligence on activities impacting a client's business” and offers advice about “appropriate corporate response strategies” [96].

The World Federation of Advertisers (WFA) has a long history of being used by the tobacco and alcohol industries in opposing advertising restrictions; for example, Peter Mitchell, the senior Guinness executive who was pivotal in the formation of the Portman Group previously led this organisation [37]. IARD has been working to further develop advertising standards and codes for the alcohol industry, a function assiduously developed by the Portman Group, whose members largely comprise the IARD member companies with a significant British presence. WFA’s work likely was in connection with these efforts.

IARD used MSL Group London to provide it with services in 2018, though the specific work conducted is unclear. The latter is a PR firm that provides a range of services, including public affairs, crisis management and media training [97].

MM Science and Advisors is a consultancy headed by ICAP and IARD’s long-standing (1996–2019) senior staff member, Marjana Martinic. Martinic had previously served as SVP Science and Policy and Deputy CEO. The consultancy claims to offer clients “accessible, comprehensive, and up-to-date insights into the health risks and benefits associated with consumer goods and help translate them into sustainable approaches” [98]. No further details are available on any specific work carried out for IARD.

## Discussion

This study aims to deepen understanding of the alcohol industry’s political activities at the global level. The study enriches understanding of alcohol industry activities through careful description and analysis of ICAP and IARD’s tax filings.

Since 1995 the major alcohol companies have worked together in ICAP, then IARD, to manage their interests at the global level, beginning when the companies themselves became globally operating entities. Through several activities, including CSR, research, and public affairs, these organisations have successfully positioned themselves as a rival source of information on alcohol-related matters to the WHO and the public health community. These activities operate as a package of inter-connected approaches developed over time to attain political goals. This study examines these operations in as much detail as is afforded by the data source. The first contribution made by this report is to open up the internal operations and strategic priorities of these organisations to external scrutiny. Both ICAP and IARD have been prominent in an era of global alcohol policy inertia in which ineffective partnerships with the industry have largely been the norm globally, contrary to the alcohol policy evidence base that stronger industry regulation is needed, particularly population-level alcohol control measures [68]. Yet IARD has evaded substantial prior scrutiny. Existing studies of ICAP have focused on materials, including toolkits, policy reports, and research, which have been tailored for public consumption [45, 99, 100]. Exclusive reliance on such data sources is potentially limiting for understanding the alcohol industry, its strategic drivers, and the nature of the threat posed to global health. Public-facing materials can be crafted in ways designed to obscure organisations’ nature, purpose, and tactics. This study is one of the few studies to make use of materials generated by ICAP/IARD staff that, while in the public domain, have a different quality from other public-facing materials. Non-profit organisations are legally required to ensure that the information provided to authorities is both accurate and verifiable. This study offers a useful vantage point for appreciating the nature of the long-term and multi-pronged public affairs program, in particular, which receives little mention in the public-facing materials.

This analysis also provides a lens through which to observe possible changes to the global alcohol industry’s strategy over time. The findings suggest ICAP’s evolution into IARD has not been accompanied by major shifts in how these organisations present themselves to tax authorities in the US, and by extension in how they operate. The activities appear marked more by continuity than by change, so it is appropriate to regard this study as offering preliminary evidence to support an understanding of IARD as an evolution of essentially the same entity as ICAP. There are, however, identifiable changes in presentation, and we in no way suggest these are trivial. Rather, this makes further study of the more visible and well-documented activities of ICAP helpful for developing the research agenda on IARD. Indeed, the success of IARD in largely evading research scrutiny provides a further reason to fill this important evidence gap and comparative analysis with ICAP is one useful frame for further analysis.

The study underscores the industry’s complex and multi-level approach to lobbying. Both ICAP and IARD tax records describe longstanding efforts to build relationships with WHO. Moreover, IARD’s involvement with public affairs firms that specialise in dealing with WHO, the UK government and EU officials provides further indications of the importance of lobbying at the global, regional and domestic level. IARD’s involvement with a UK Government agency is striking. Further research will be required to identify the nature of those interactions, and the implications for the effectiveness of lobbying in other national and international contexts.

The nature of the tax filings means that there are limitations to how much can be inferred about ICAP and IARD’s activities, whilst offering a novel perspective on the internal functioning of these entities. First, an analysis of ICAP and IARD’s materials helps provide new insights into the goals and activities of these organisations, for example, on public affairs, but by design, it cannot tell us about the effectiveness of efforts. Second, although the tax fillings included key expenditure data, the study’s capacity for quantitative assessment is limited by the nature of the dataset. The IRS implemented several changes to Form 990 during the years in question, making it difficult or unwise to evaluate changes between specific program expenditures year-to-year. As such, we largely restricted analytic attention to the qualitative description of activities and aggregate spending. The result is a potentially incomplete account of program and expenditure changes. Third, the study did not include tax records from 2005–2010, when there was major activity at the global level. In 2010, the WHO released its Global Strategy to Reduce the Harmful Use of Alcohol, following years of consultations with stakeholders, including the alcohol industry [101]. Future research, however, could draw on the tax records that were omitted as well as ICAP submissions to that process. Fourth, the goals of the study were descriptive in nature; the data do not permit us to provide causal explanations for why IARD and ICAP function the way that they do. Addressing questions of this nature would require additional data sources, including interviews with key personnel and/or access to internal documents that are not presently available. Finally, this study does not engage with the scientific content produced by ICAP or IARD. Between 1998 and 2010, ICAP published several research and policy materials, which helped frame key public health debates [45, 99, 102]. While studies analysing the scientific content of ICAP or IARD’s materials are vital, this present study is confined to the organisational units and processes.

## Conclusion

In the interest of global health, researchers and policymakers require a deeper understanding of how the alcohol industry operates as a political actor. Industry has long been seen as a legitimate participant in policymaking [9]. There are some indications that change may be afoot in this respect. WHO leaders, for example, have expressed concerns about industry involvement in policymaking [103]. Furthermore, WHO staff have been advised not to engage with the alcohol industry on certain matters [104]. Yet, the alcohol industry’s approach to influencing policymaking is far from restricted to lobbying policymakers. Industry has several other tools at its disposal, including mobilising other interests during policy disputes (i.e. proxies) [105], venue shopping [106–108], and influencing the scientific content that underpins policy debates [46, 109]. The sophisticated quality of these strategies, and indeed the nature of organisations such as ICAP and IARD, suggests that public health interests need to thoroughly examine the significance of the alcohol industry’s activities, particularly in relation to influencing the public and public policy.

## Data Availability

All data generated or analysed during this study are included in this published article or in the hyperlinks provided.

## Abbreviations

CSR: Corporate social responsibility
GAHD: Global Actions on Harmful Drinking
IARD: International Alliance for Responsible Drinking
IPAC: International Center for Alcohol Policies
IRS: Internal Revenue Service
SAPRO: Social Aspects Public Relations Organisations
UCI: Unhealthy commodity industries
WHO: World Health Organization
WFA: World Federation of Advertisers
